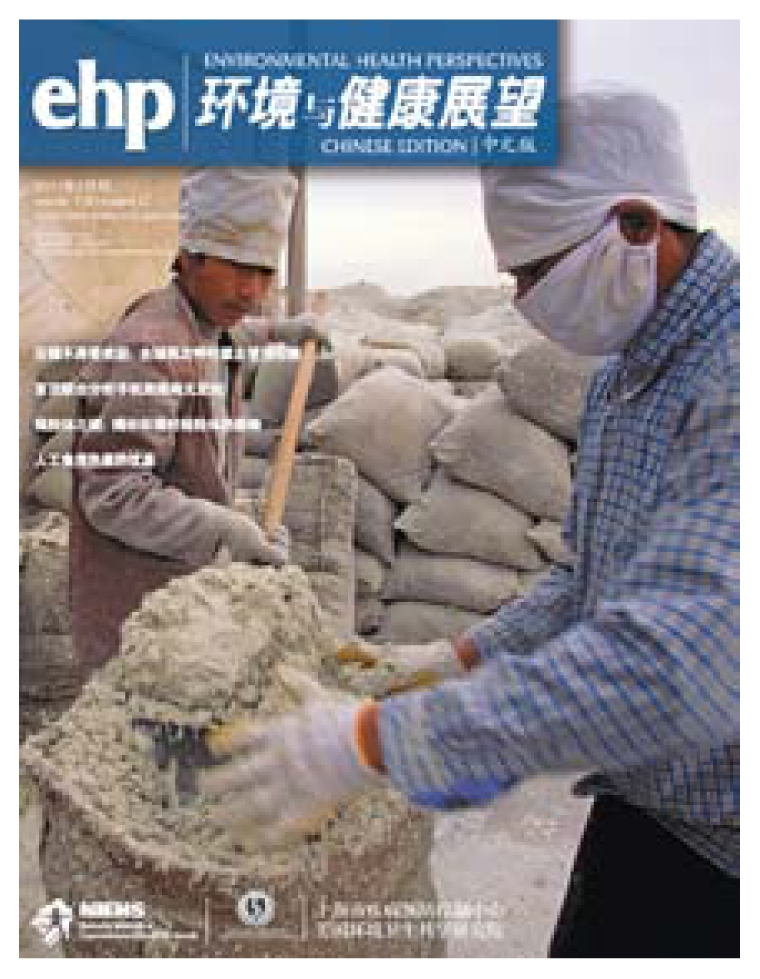# Editor’s Note

**Published:** 2011-04

## EHP Chinese Edition

Ten years ago, *Environmental Health Perspectives* (*EHP*) and the Shanghai Municipal Center for Disease and Prevention (SCDC) launched a Chinese edition of the journal to be published quarterly. *EHP* Chinese Edition consists of selected news articles taken from the English version of the journal and translated into Chinese, as well as editorials and commentaries written by scientists whose primary language is Chinese. We have been encouraged by the fact that interest in *EHP* Chinese Edition from students, researchers, and policy makers has grown significantly over the past several years. In response, a digital version of *EHP* Chinese Edition was launched in 2010 to make it available to more readers. A decision was also made to publish *EHP* Chinese Edition bimonthly starting in February of this year. In addition, a new Editorial Review Board was formed to help evaluate content in the journal and develop strategic goals and objectives for future growth. We are excited about the opportunity to strengthen our collaboration with the SCDP and provide *EHP* Chinese Edition on a more frequent basis to the Chinese-speaking community.

## Figures and Tables

**Figure:**